# Supplementary material for: Effects of Androgen Treatment on Growth in Patients with 5-α-Reductase Type 2 Deficiency
Source: J Pers Med. 2023 Jun 13;13(6):992. doi: 10.3390/jpm13060992 (PMC10301148; doi:10.3390/jpm13060992)
Supplement: Supplementary file 1 [file jpm-13-00992-s001.zip › jpm-2394763-supplementary.pdf]

FIGURE S1 Chromatogram Results of Sanger Sequencing Analysis for Patient 12 and Patient 14.
A, Patient No. 12

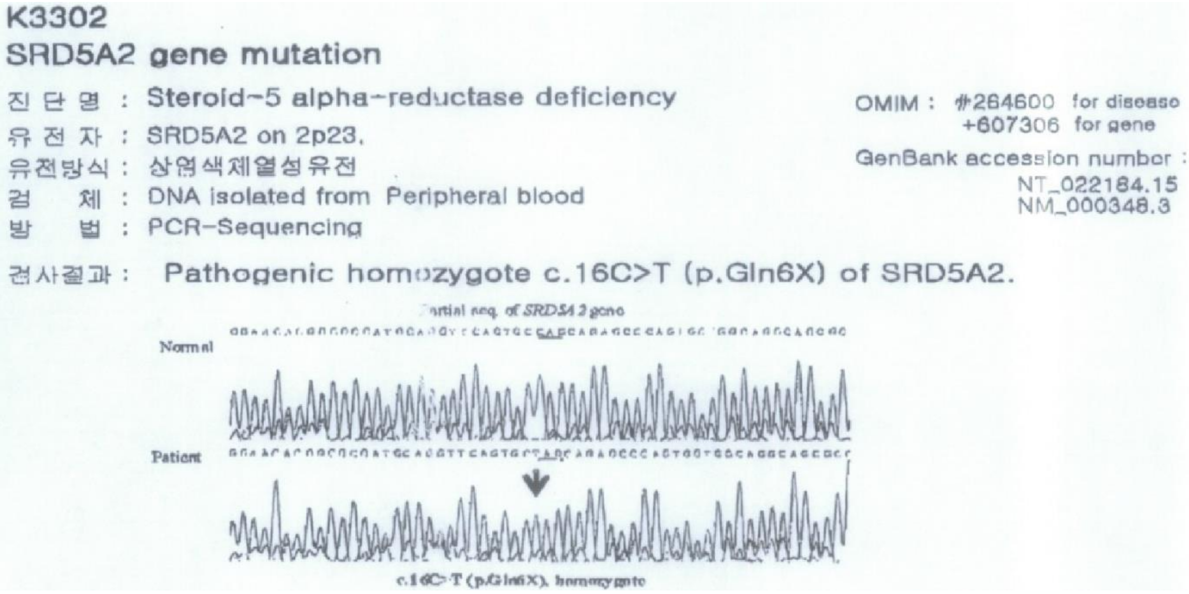

B, Patient No. 14

검체번호	1411279340
검사일	2014-07-30
의뢰의	[redacted]
Reason for Test	Pseudovaginal perineoscrotal hypospadias

진단명

진단명
Micropenis

Origin of sample	Peripheral Blood
Used DNA Marker	SRD5A2
Test method	Direct sequencing

Result

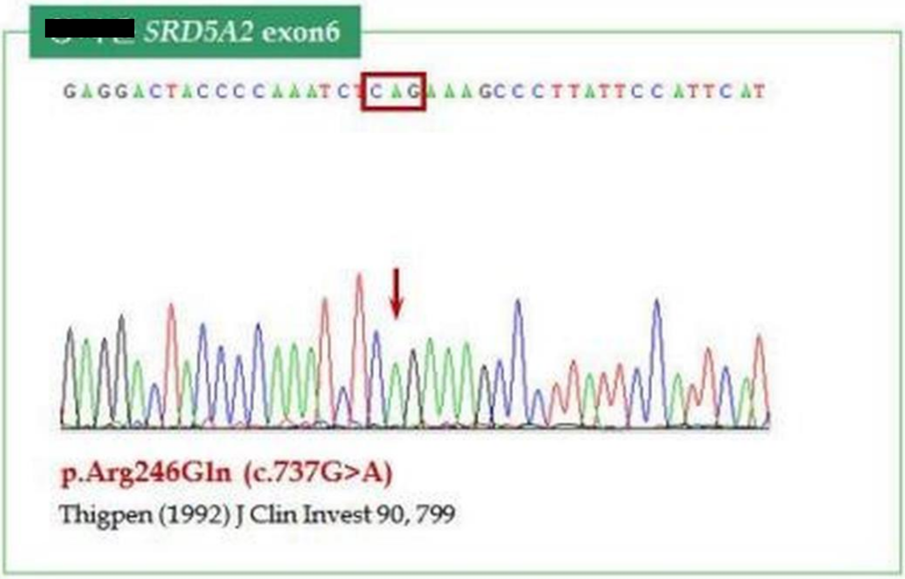

Interpretation	본 검사에서는 Pseudovaginal perineoscrotal hypospadias의 원인으로 알져진 SRD5A2 유전자의 돌연변이 여부를 direct sequencing 방법을 통하여 확인하였습니다. 검사결과 SRD5A2 유전자의 exon6에서 p.Arg246Gln 돌연변이가 homozygote 형태로 발견되었습니다. 문헌에 의하면 이와 같은 돌연변이가 Pseudovaginal perineoscrotal hypospadias를 유발하는 것으로 알려져 있으며 빈도가 매우 낮은 polymorphism으로도 알려져 있습니다.(G/A=0.3%, 662 european) 따라서 환자는 SRD5A2 유전자의 돌연변이로 인한 Pseudovaginal perineoscrotal hypospadias 일 가능성이 높으며 정확한 결과를 위하여 가족에 대한 검사도 진행 해 보시기 바랍니다.
Date of report	2014-08-20
판독의	이진성

TABLE S1 Molecular data of 19 patients with 5-α-reductase type 2 deficiency.

Patient No.	*SRD5A2* mutation (NM_000348.4)							
	Allele 1				Allele 2			
	Nucleotide	Amino acid	Interpretation†	Variation ID	Nucleotide	Amino acid	Interpretation†	Variation ID
1	c.737G>A	R246Q	P	459645	c.737G>A	R246Q	P	459645
2	c.737G>A	R246Q	P	459645	c.16C>T	Q6X	P	436859
3	c.737G>A	R246Q	P	459645	c.655del	F219fs	P	97381
4	c.737G>A	R246Q	P	459645	c.606G>A	G203S	P	459640
5	c.737G>A	R246Q	P	459645	c.606G>A	G203S	P	459640
6	c.737G>A	R246Q	P	459645	c.735C>T	R246W	P	3337
7	c.679G>A	R227Q	LP	3351	c.737G>A	R246Q	P	459645
8	c.679G>A	R227Q	LP	3351	c.192G>C	A65P	LP	988403
9	c.679G>A	R227Q	LP	3351	c.157T>C	W53R	LP‡	–
10	c.737G>A	R246Q	P	459645	c.737G>A	R246Q	P	459645
11	c.737G>A	R246Q	P	459645	c.737G>A	R246Q	P	459645
12	c.16C>T	Q6X	P	436859	c.16C>T	Q6X	P	436859
13	c.679G>A	R227Q	LP	3351	c.16C>T	Q6X	P	436859
14	c.737G>A	R246Q	P	459645	c.737G>A	R246Q	P	459645
15	c.679G>A	R227Q	LP	3351	c.16C>T	Q6X	P	436859
16	c.679G>A	R227Q	LP	3351	c.737G>A	R246Q	P	459645
17	c.679G>A	R227Q	LP	3351	c.737G>A	R246Q	P	459645
18	c.679G>A	R227Q	LP	3351	c.679G>A	R227Q	LP	3351
19	c.679G>A	R227Q	LP	3351	c.737G>A	R246Q	P	459645

Abbreviations: P, pathogenic; LP, likely pathogenic. †, The interpretations were evaluated according to the American College of Medical Genetics and Genomics (ACMG) guideline. ‡, According to the ACMG guideline, the variant was classified as likely pathogenic based on the following evidence: PM1, PM2, PP3, and PP4.

TABLE S2 Age at treatment initiation, type of androgen used, dose and duration of treatment, and its effects on patients who received androgen treatment.

Patient No.	Cycle	Age	Type	Dose	Duration	Effect
8	8-1	5.6 y	DHT	1 dose	1 month	Good
	8-2	11.6 y	DHT	1 dose	1 month	Good
	8-3	14.1 y	DHT	1 dose	3 months	Mild to moderate
		14.4 y	DHT	2 doses	1.5 months	Good
9	9-1	1.5 y	DHT	1 dose	1 month	No
		1.6 y	DHT	2 doses	1.5 months	Good
	9-2	2.9 y	DHT	1 dose	2 months	Mild
		3.5 y	DHT	1 dose	2 months	No
		3.6 y	DHT	2 doses	5 months	Good
10	10-1	0.3 y	DHT	1 dose	4 months	Good
		1.0 y	DHT	1 dose	2 months	Good
		1.6 y	DHT	1 dose	1.5 months	Good
	10-2	3.2 y	DHT	1 dose	1 month	Moderate
11	11-1	0.8 y	DHT	1 dose	3 months	Good
12	12-1	0.6 y	DHT	1 dose	0.5 month	Good
		1.5 y	DHT	1 dose	1 month	Moderate
		2.1 y	DHT	1 dose	2 months	Moderate
	12-2	3.2 y	DHT	1 dose	0.5 month	Moderate
		3.7 y	DHT	1 dose	1 month	Moderate
		4.2 y	DHT	1 dose	1 month	Moderate
	12-3	8.6 y	DHT	2 doses	1 month	Moderate
		9.2 y	DHT	2 doses	1 month	Moderate
13	13-1	1.5 y	DHT	2 doses	3 months	Good
		2.4 y	DHT	2 doses	0.5 month	Good
	13-2	6.5 y	TE	25 mg Q4W	3 times	Good
	13-3	9.4 y	TE	25 mg Q3W	5 times	Moderate
14	14-1	0.8 y	DHT	1 dose	1 month	Moderate
		1.4 y	TE	37.5 mg	1 time	No
	14-2	2.9 y	TE	50 mg	1 time	No
		3.0 y	DHT	1 dose	1.5 months	Mild
		3.6 y	DHT	1 dose	1 month	Mild
		3.7 y	DHT	1.5 doses	2.5 months	Mild
	14-3	6.1 y	DHT	1.5 doses	3 months	Moderate
		6.3 y	DHT	3 doses	0.5 month	Moderate
	14-4	7.6 y	DHT	2 doses	1.5 months	Mild
		8.2 y	DHT	2 doses	3 months	Mild
15	15-1	21 day	TE	25 mg	1 time	Mild
		35 day	TE	37.5 mg	1 time	Mild
		49 day	TE	50 mg	1 time	Mild
		0.5 y	DHT	1 dose	1 month	Good
		0.7 y	DHT	1 dose	1 month	Good
16	16-1	0.7 y	TE	100 mg	1 time	Good
	16-2	2.5 y	DHT	1 dose	2 months	Good
	16-3	6.8 y	TE	50 mg Q3W	4 times	No
17	17-1	10.3 y	TE	50 mg Q2W	2 times	Moderate
	17-2	11.5 y	TE	150 mg	1 time	Mild to moderate
18	18-1	13.2 y	TE	50 mg Q3W	4 times	Mild
	18-2	15.8 y	TE	150mg Q3W	3 times	Mild
19	19-1	10.0 y	TE	250 mg	1 time	Good

Abbreviations: DHT, dihydrotestosterone; TE, testosterone enanthate; Q4W, once every 4 weeks; Q3W, once every 3 weeks; Q2W, once every 2 weeks
